# Detection, Genomic Characterization, and Antibiotic Susceptibility of *Salmonella* Anatum SPBM3 Isolated from Plant-Based Meat

**DOI:** 10.3390/foods14213710

**Published:** 2025-10-30

**Authors:** Phatchara Phayakka, Kitiya Vongkamjan, Pacharapong Khrongsee, Kuttichantran Subramaniam, Auemphon Mordmueng, Wattana Pelyuntha

**Affiliations:** 1 School of Education, Walailak University, Nakhon Si Thammarat 80160, Thailand; phatchara.ph@mail.wu.ac.th; 2 Department of Biotechnology, Faculty of Agro-Industry, Kasetsart University, Bangkok 10900, Thailand; kitiyavongkamjan.a@ku.th; 3 Department of Infectious Diseases and Immunology, College of Veterinary Medicine, University of Florida, Gainesville, FL 32610, USA; firstpachar@ufl.edu (P.K.); kuttichantran@ufl.edu (K.S.); 4 Emerging Pathogens Institute, University of Florida, Gainesville, FL 32610, USA; 5 Faculty of Veterinary Science, Prince of Songkla University, Songkhla 90110, Thailand; 6 School of Medicine, Walailak University, Nakhon Si Thammarat 80160, Thailand; auemphon.mo@wu.ac.th; 7 Futuristic Science Research Center, School of Science, Walailak University, Nakhon Si Thammarat 80160, Thailand; 8 Research Center for Theoretical Simulation and Applied Research in Bioscience and Sensing, Walailak University, Nakhon Si Thammarat 80160, Thailand

**Keywords:** antibiotic resistance, genome analysis, MLST, plant-based meat, *Salmonella*

## Abstract

Plant-based meat (PBM) products have rapidly grown in popularity due to increasing consumer demand for sustainable, ethical, and health-oriented food alternatives. However, these novel products may pose microbiological risks similar to traditional meats, including contamination by *Salmonella* spp. In this study, PBM samples (*n* = 63), including raw products (ground pork, mushroom, and burger) and cooked products (chicken tender, chicken breast, nugget, and beef), were collected from local retail markets in Bangkok, Thailand. The prevalence of *Salmonella* spp. was assessed by calculating the proportion of confirmed positive samples relative to the total number of PBM products tested. Additionally, the genomic characteristics and antibiotic susceptibility of *Salmonella* isolated from PBM were also investigated. From the result, *Salmonella enterica* was detected in 2.44% (1/41) of raw PBM samples, whereas no contamination was observed in cooked PBM products (0/22). Serovar identification revealed the isolate to be *S.* Anatum. Whole genome sequencing (WGS) analysis revealed the genome of *S.* Anatum SPBM3 consisted of 4,726,256 base pairs with 52.15% GC content, encoding 4717 coding sequences (CDS). Pangenomic analyses placed *S.* Anatum SPBM3 within a distinct sub-cluster closely related to pathogenic *Salmonella* strains previously reported, confirming its identity as part of the *S. enterica* lineage. The genome harbored 67 antimicrobial resistance genes, 5 prophage elements, and 305 key virulence determinants. Phenotypically, the isolate exhibited susceptibility to most tested antibiotics but showed intermediate resistance to streptomycin, ciprofloxacin, and colistin. Our findings highlight the potential microbial risks associated with PBM products and emphasize the importance of genomic surveillance to ensure food safety and public health protection as dietary preferences evolve toward non-traditional food matrices.

## 1. Introduction

Plant-based meat (PBM) products have emerged as a transformative innovation in the global food industry, primarily driven by growing consumer demand for sustainable, ethical, and health-conscious alternatives to conventional meat [1,2]. These products are engineered to mimic the taste, texture, and nutritional composition of animal-derived meats through the utilization of plant-based ingredients such as soy, pea protein, and wheat gluten [3]. Although frequently perceived as healthier and more environmentally friendly, PBM products are not inherently free from microbial risks [4]. Their complex production processes, encompassing ingredient sourcing, formulation, and packaging, introduce multiple critical control points where microbial contamination can occur, emphasizing the need for stringent safety assessments [5].

Foodborne illnesses caused by *Salmonella* spp. and other pathogens remain a persistent global public health concern, with the World Health Organization (WHO) estimating millions of cases annually [6,7]. Among the diverse *Salmonella* serovars, *S.* Anatum has been frequently associated with foodborne outbreaks, posing significant threats to human health and the integrity of food safety systems [8]. Traditionally, *Salmonella* contamination has been predominantly linked to animal-derived products such as poultry, eggs, and dairy [9]. However, as consumer dietary preferences shift toward plant-based foods, new microbiological risks emerge, challenging existing food safety paradigms [10]. Although PBM are designed to emulate conventional meat products, they are still susceptible to microbial contamination, including pathogens like *Salmonella*, which may infiltrate the supply chain through raw ingredients, contaminated processing environments, or cross-contamination during packaging and distribution [4,5]. Therefore, investigating the genomic characteristics of *Salmonella* from PBM products is crucial for accurately assessing their potential risks and for the development of robust food safety strategies.

Recent advancements in genomic technologies, particularly whole genome sequencing (WGS), have revolutionized the study of foodborne pathogens by providing high-resolution insights into their genetic architecture, including antimicrobial resistance (AMR), virulence determinants, and evolutionary dynamics [11,12]. WGS has become a cornerstone in epidemiological surveillance and outbreak investigation, enabling precise tracking of pathogen sources and transmission pathways [13]. Despite these advances, a substantial knowledge gap remains concerning the genomic profiles of *S*. Anatum isolated from PBM products, as most genomic studies to date have focused on traditional animal-derived food matrices [10]. This scarcity of data limits current understanding of *Salmonella* risks associated with emerging plant-based alternatives and hinders the development of targeted interventions to mitigate potential outbreaks.

To address the potential microbiological risks in PBM products, where the occurrence of *Salmonella* contamination has been considered rare and scarcely documented, this study investigated an isolate obtained from PBM samples collected from local retail markets in Bangkok, Thailand. To our knowledge, this represents one of the first reports of *Salmonella* detection in PBM. WGS was employed to characterize the isolate by analyzing antimicrobial resistance genes, virulence factors, and phylogenetic relationships. By generating such genomic data, this study aims to provide an essential foundation for food safety monitoring and to inform the development of preventive strategies to mitigate contamination risks as plant-based diets continue to expand globally.

## 2. Materials and Methods

### 2.1. Plant-Based Meat Collection and Isolation of Salmonella

Raw and cooked PBM products (*n* = 63) were collected from local retail markets at Chatuchak, Bangkok, Thailand. All collected products were certified PBM, produced by commercial manufacturers, and labeled as vegan/vegetarian on the packaging. These included ground pork analogs, chicken-style tenders, chicken-style breast filets, nuggets, burger patties, and beef-style strips. The primary protein sources were soy, pea, and/or mushroom with additional ingredients such as wheat gluten, vegetable oils (coconut, canola), and flavoring agents to mimic animal meat texture and taste. Detailed information on the composition of each PBM product, including primary protein sources and other key ingredients, is provided in Table S1. None of the products contained animal-derived ingredients according to the manufacturer’s labels.

For *Salmonella* detection, twenty-five grams of each sample were processed for *Salmonella* isolation by adding 225 mL of buffered peptone water (BPW) supplemented with *Salmonella* Supp Tab (#421202, Biomérieux, Marcy-l’Étoile, France) and rigorously blended with a circulator lab blender (Seward Stomacher^®^ Model 400, Seward^TM^, West Sussex, UK). The mixture was further incubated at 41.5 °C for 24 h. Then, a loopful of culture fluid was further streaked on the SALMA^®^ agar plate (Biomérieux, Marcy-l’Étoile, France) according to the modified ISO 6579-1: 2017 [14], provided by the Biomérieux company.

The typical purple colonies on the SALMA^®^ agar were observed and recorded. Biochemical tests, including the urease, lysine iron sugar (LIA), and triple sugar iron (TSI) tests, were used for *Salmonella* confirmation according to the ISO 6579-1: 2017 recommendation. Suspected isolates of *Salmonella* were further confirmed via latex agglutination test by a commercial service company (S&A Reagents Lab. Ltd., Part., Bangkok, Thailand). The prevalence of *Salmonella* in all samples was reported. The study was conducted following the biosafety regulations and was approved by the Institutional Biosafety Committee of Walailak University (Ref. No. WU-IBC-67-070).

### 2.2. DNA Extraction and Sequencing

Genomic DNA was extracted from a single bacterial colony using the QIAamp UCP Pathogen Mini Kit (QIAGEN, Hilden, Germany), following the manufacturer’s protocol. The quality and quantity of the extracted DNA was assessed using a NanoDrop™ 2000 spectrophotometer (Thermo Scientific™, Waltham, MA, USA). The DNA extract was then submitted to Biomarker Technologies (BMK) GmbH (Münster, Germany) for WGS on the Illumina NovaSeq platform with paired-end 150 bp (PE150) reads.

### 2.3. Genome Quality, Assembly, and Analysis

The quality of sequencing reads was assessed using FastQC v0.11.9 [15]. Adapter sequences and low-quality bases were trimmed using Fastp v0.24.0 with a Phred quality score threshold of 20 to ensure high-quality reads [16]. After quality control and trimming, de novo assembly of the remaining paired-end reads was performed using CLC Genomics Workbench v20.0.4, with default parameters. Assembled contigs were filtered to include only those with coverage greater than 100× and were then selected for further analysis. Draft genome assembly was submitted to the TCS in JSpeciesWS for taxonomic identification and compared against the GenomesDB reference database (https://jspecies.ribohost.com/jspeciesws/, accessed on 17 July 2025) [17].

Sequence typing was performed using the PubMLST platform (https://pubmlst.org) [18], and the genome was annotated using Prokka v1.14.6 with default parameters [19]. Subsequently, the pangenome analysis was conducted using Roary v3.12.0 on 220 NCBI RefSeq assembly files of *S. enterica* sequence type 64 (ST64) genomes, *S.* Anatum SPBM3 (from the current study), and *S. enterica* subsp. *enterica* serovar Oranienburg strain CFSAN039538 as an outgroup, which was detailed in Supplemental Data S1 [20]. A maximum likelihood (ML) phylogeny, based on the concatenated core gene alignment generated by Roary, was generated using IQ-Tree v1.6.12 with 1000 replicates of ultrafast bootstrap to determine branch support [21]. Finally, the ML tree was visualized using iTol-v6 [22]. Prophage regions were detected using the PHASTEST web server (https://phastest.ca/) [23,24,25]. To identify virulence, antibiotic-resistant genes, the draft genome was analyzed using the Genome Annotation services via the Bacterial and Viral Bioinformatics Resource Center (BV-BRC) [26]. Plasmid content was identified using the PlasmidFinder 2.0 server, also hosted by the Center for Genomic Epidemiology [27]. This Whole Genome Shotgun project has been deposited at DDBJ/ENA/GenBank under the accession JBQJIX000000000.

### 2.4. Antibiotic Susceptibility Test

Antibiotic susceptibility testing (AST) was performed using the Kirby–Bauer disk diffusion method following CLSI 2024 guidelines [28]. Briefly, isolated colonies from overnight cultures were suspended in sterile saline to match a 0.5 McFarland standard (≈1.5 × 10^8^ CFU/mL). A sterile swab was dipped into the suspension, and after removing the excess fluid, the entire surface of Mueller–Hinton Agar (MHA) plates (4 mm thickness) was evenly inoculated by streaking in three directions. After drying for 3–5 min, 13 selected antibiotic disks, including ampicillin (10 µg), amoxicillin-clavulanic acid (30 µg), gentamicin (10 µg), streptomycin (10 µg), ceftriaxone (30 µg), tetracycline (30 µg), ciprofloxacin (5 µg), nalidixic acid (30 µg), trimethoprim-sulfamethoxazole (1.25/23.75 µg), chloramphenicol (30 µg), colistin (10 µg), tobramycin (10 µg), and amikacin (30 µg) were applied. Plates were incubated at 35 °C for 16–18 h. After incubation, zones of inhibition were measured in millimeters and interpreted as susceptible (S), intermediate (I), or resistant (R) according to CLSI 2024 breakpoints. Quality control was performed using *Escherichia coli* ATCC 25922 as the reference strain, in accordance with CLSI 2024 guidelines, to validate the accuracy of the disk diffusion assay.

## 3. Result and Discussion

### 3.1. Prevalence of Salmonella in Collected Plant-Based Meat Products

The prevalence of *Salmonella* in PBM products collected from local retail markets in Bangkok was 1.59% (1/63). Specifically, *Salmonella* was detected in 3.57% (1/28) of raw plant-based ground pork analog samples, while none of the cooked samples tested positive (0/22) (Table 1). Further identification using the latex agglutination test confirmed that the isolate was *S.* Anatum and named SPBM3.

The prevalence of *Salmonella* in PBM products appears to be relatively low compared to traditional meat products. However, one study indicated that PBM could serve as vectors for transmitting certain foodborne pathogens [29]. Our findings of a 1.59% overall *Salmonella* prevalence in PBM products from Bangkok retail markets, with 2.44% in raw samples and 0% in cooked samples, provide valuable insights into the potential food safety risks associated with these products. A comprehensive 3-year targeted survey conducted by the Canadian Food Inspection Agency analyzed 1026 samples of ready-to-eat non-soy PBM alternatives and found no *Salmonella* contamination (0%) in any of the samples [30]. This result contrasts with the higher prevalence rates previously reported in China for conventional meat products, where an overall prevalence of 19.7% was observed in retail meat samples, with pork showing the highest contamination rate at 37.3% [31]. In the Philippines, 57.64% of collected meat samples were reported to be contaminated with *Salmonella* [32]. While specific data on *Salmonella* serovars in PBM products is limited, studies on conventional meat products have identified *S.* Derby and *S.* Typhimurium as the most prevalent serovars, accounting for 46.3% of all strains in Chinese retail meats [31]. The prevalence of *Salmonella* in traditional poultry meat products varies by region, with some areas reporting rates as high as 44.3% (95% CI: 29.9–59.7%) in Shaanxi, China [33]. However, it is essential to note that PBM may introduce new pathogen risks not commonly associated with animal meats, such as *Bacillus cereus*, due to the inclusion of grain-based ingredients, soy and pea proteins, and food starches. As the PBM industry continues to grow, more specific studies focusing on *Salmonella* prevalence and serovars in these products will be necessary to ensure food safety and inform regulatory requirements.

In our study, a limitation is the relatively small sample size (*n* = 63) and the fact that all PBM products were collected from retail markets in a single metropolitan area (Bangkok). These factors may restrict the representativeness and statistical power of the results. Therefore, future investigations should expand the number of samples and diversify the sampling locations to obtain a more comprehensive assessment of *Salmonella* contamination risks in PBM across different regions.

### 3.2. Genome Quality, Assembly, and Analysis

The WGS produced a total of 18,669,958 reads, which assembled into 30 high-quality contigs after filtering for a minimum contig size of 500 bp and a minimum coverage of 100×. The final genome assembly comprised 4,726,256 bp, encoding 4717 predicted coding sequences (CDS) with an average GC content of 52.15%. In addition, there was no plasmid found within the genome. The analysis of Average Nucleotide Identity (ANIb) of the selected contigs, performed with JSpeciesWS showed 99.94% nucleotide identity to *S.* Anatum (Table S2).

**Figure 1:**
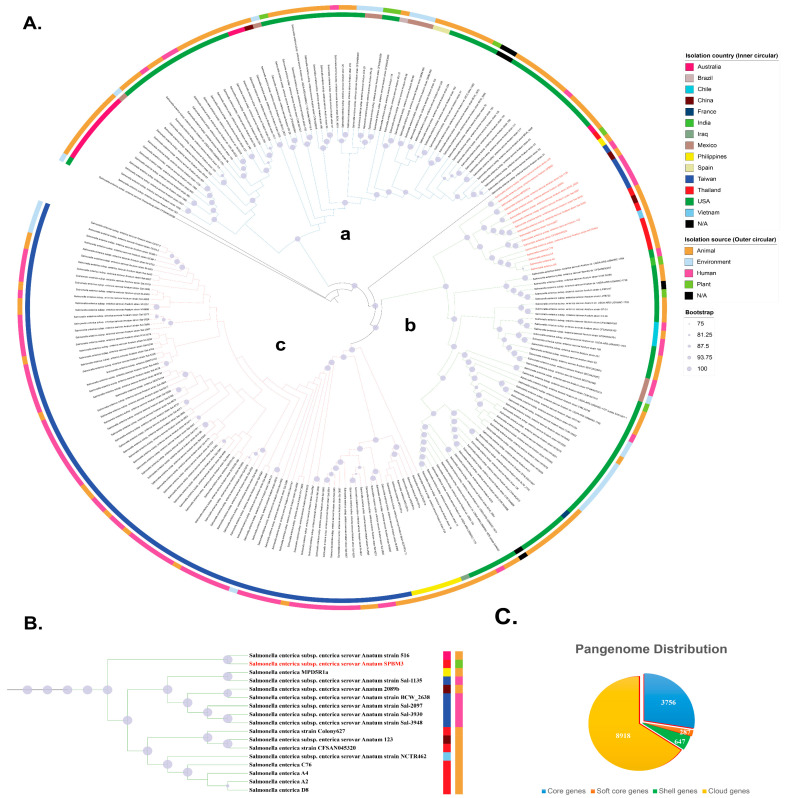
Pangenome analysis of *S. enterica* ST64 genomes (*n* = 221) and *S.* Oranienburg strain CFSAN039538 as an outgroup. (**A**) Maximum-likelihood phylogenetic tree constructed from the core genome sequences of ST64 isolates. Node size corresponds to bootstrap support (Only values ≥75% are shown), with larger nodes indicating higher boostrap values. Inner and outer color bands represent the country of isolation and source type, respectively. The phylogeny is divided into three major clades: (a) isolates from Australia and the Americas, (b) a mixture of isolates from Western countries and Asia, and (c) isolates from Taiwan and the Philippines. The position of *S. enterica* serovar Anatum strain SPBM3 is highlighted in red. (**B**) Enlarged view of the clade containing serovar Anatum SPBM3, showing close relationships with isolates from Australia, China, the Philippines, Thailand, Taiwan, and Vietnam. Notably, SPBM3 clustered with human gastroenteritis strains isolated from Taiwan. Colored squares adjacent to strain names represent the isolation country (inner squares) and source type (outer squares), consistent with panel (**A**). (**C**) Distribution of the pangenome among the analyzed isolates, showing proportions of core genes, soft-core genes, shell genes, and cloud genes.

The identification of *S.* Anatum SPBM3 from the PBM sample as ST64 presents a significant food safety concern, particularly as ST64 is a globally disseminated lineage frequently linked to foodborne outbreaks [34]. Pangenome analysis of 221 ST64 *S. enterica* genomes revealed a total of 13,608 genes, illustrating large genomic diversity within this lineage (Figure 1C). Of these, the core genome—defined as genes present in ≥99% of strains—comprised 3756 genes (28% of the total gene pool). An additional 287 soft-core genes (present in ≥95% but <99% of strains) accounted for ~2%. The majority of genomic variability arose from accessory genes, which represented 70% of the pangenome. These included 647 shell genes (present in ≥15% but <95% of strains), which accounted for ~5%, and 8918 cloud genes (present in <15% of strains), which accounted for ~65%. This large accessory gene repertoire showed the high genomic plasticity of ST64 and its potential to adapt to a variety of ecological niches, including diverse hosts and food matrices [35]. The predominance of variable gene content among ST64 strains suggests a capacity for rapid adaptation, antimicrobial resistance acquisition, and host specialization, traits that likely enhance survival across environments ranging from animal reservoirs to plant-based food products. Such diversity may also contribute to the emergence of strains with increased virulence or environmental persistence, further complicating outbreak detection and control efforts.

A maximum likelihood (ML) phylogeny, based on the concatenated core gene alignment supported that the phylogeny is divided into three major clades: (a) isolates from Australia and the Americas, (b) a mixture of isolates from Western countries and Asia, and (c) isolates from Taiwan and the Philippines (Figure 1A). Interestingly, clade (b) contained mixture of continental isolates spanning both the Eastern and Western hemispheres, suggesting that cross-border dissemination of ST64 has increasingly facilitated global movement of this lineage. Within clade (b), the subclade containing SPBM3 was largely composed of animal- and human-associated isolates, clustering with strains from Australia, China, the Philippines, Thailand, Taiwan, and Vietnam. This group also included all previously reported Thai ST64 isolates recovered from animal products, suggesting the source of contamination may come from a local strain that is distributed in the Southeast Asia region. Notably, SPBM3 clustered closely with human gastroenteritis strains (Sal-1135, 2097, 3930, and 3948) previously isolated from patients at Chang Gung Memorial Hospital in Taiwan (Figure 1B), raising the possibility that SPBM3 harbors virulence traits capable of causing human Salmonellosis. This is particularly concerning given that SPBM3 originated from a PBM analog, a product type often promoted as a safer and more sustainable alternative to conventional meat. Recent studies have reported the presence of zoonotic and opportunistic pathogens in plant-based foods, likely introduced through cross-contamination during harvesting, processing, or storage [36,37]. These findings challenge assumptions regarding the inherent safety of alternative protein sources and highlight the importance of comprehensive food safety protocols tailored to novel food categories at every node of the supply chain [38].

It should be noted that the detection of *Salmonella* in raw PBM does not necessarily indicate a direct risk to consumers if the product is properly cooked, as thermal processing would inactivate the pathogen. Nevertheless, the presence of *Salmonella* at the raw stage is still a relevant food safety concern because of the potential for cross-contamination to ready-to-eat foods or food-contact surfaces during handling. Moreover, although our genomic characterization revealed the presence of multiple virulence determinants, no pathogenicity assays were performed; therefore, the clinical significance of this isolate cannot be inferred. Our results should thus be interpreted as an indication of the need for continued surveillance rather than direct evidence of disease risk.

Nonetheless, it should be noted that this study was based on a single isolate (*S.* Anatum SPBM3). Without additional isolates or systematic sampling from raw materials, processing environments, or food handlers, the representativeness of SPBM3 and the precise route of contamination cannot be determined. This limitation reduces the ability to generalize our findings but underscores the importance of future large-scale studies that include multiple isolates and environmental tracking to better inform food safety strategies in the PBM sector.

The findings reinforce the need to integrate WGS into routine food safety surveillance. While countries like Thailand have begun implementing WGS to trace *Salmonella* outbreaks and monitor antimicrobial resistance [39,40,41], the inclusion of plant-based foods in these programs remains insufficient. Given the growing global demand for plant-based diets, it is essential to extend surveillance to this sector. Moreover, a One Health approach, connecting human, animal, and environmental health, should be adopted to effectively mitigate the risks posed by zoonotic pathogens entering novel food systems [42].

**Figure 2:**
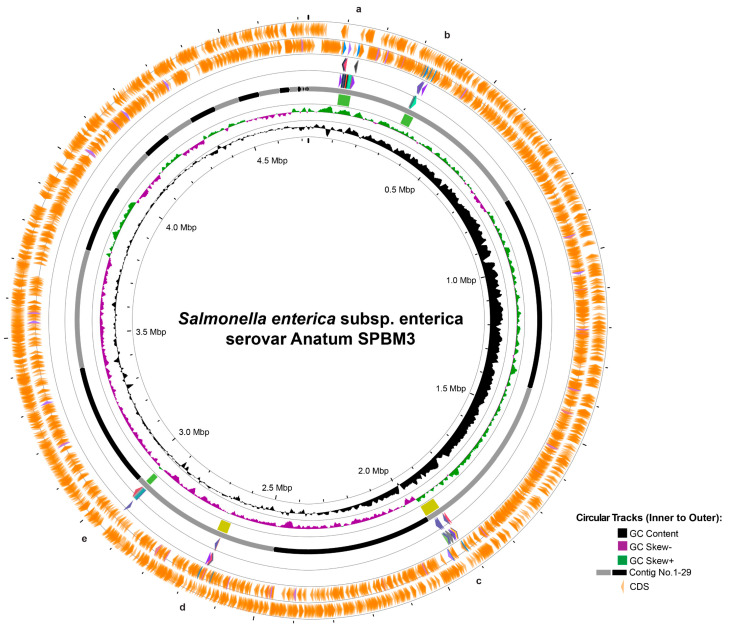
Circular genome visualization and prophage prediction of *Salmonella* Anatum SPBM3. The circular representation displays genomic features from innermost to outermost rings: GC content (black), GC skew negative (purple), GC skew positive (green), contig boundaries numbered from 1 to 30 (gray and black segments), and predicted coding sequences (CDS, orange arrows). Marked regions (a–e) correspond to predicted prophage elements identified in the genome.

Genome annotation of *S.* Anatum SPBM3 revealed the presence of 305 putative virulence genes (Table S3), highlighting its potential pathogenicity. The most frequently identified genes belong to the metabolism-related group, including *aroA*, *citC*, *acnA*, and *ilvG*, which are involved in pathways such as the shikimate pathway and the TCA cycle. These genes are not only essential for bacterial growth but also play a crucial role in survival under nutrient-limited conditions, such as within host immune cells [43,44,45,46]. Besides that, they are also associated with the biosynthesis of precursors for lipopolysaccharides (LPS), aminoglycosides, and various immune-modulating compounds. Notably, several key determinants commonly associated with *Salmonella* pathogenesis were also identified, including components of the type III secretion system (T3SS) such as *invA*, *sipD*, *spiA*, *spiC*, *avrA*, and *siiD*, which are essential for host cell invasion and immune modulation. In addition, genes linked to biofilm formation (*csgA*, *csgB*), fimbrial adherence (*fimC*, *fimF*, *fimH*), and flagellar biosynthesis were also detected (Figure 2). Additionally, key regulatory genes, such as *arcA* and *phoP*, were also identified. These are part of two-component regulatory systems that enable the bacteria to adapt to environmental conditions such as acidity, low magnesium concentration, or destruction by macrophages. There are also genes directly involved in immune evasion, such as *sodC* (superoxide dismutase), *rfaH* (involved in LPS biosynthesis), and *vacB* (ribonuclease RNase R), which help the bacterium evade destruction by macrophages and neutrophils. These highlight the bacterium’s potential for survival within the gastrointestinal tract and inside host immune cells. These virulence factors, widely reported in both human and animal isolates, contribute to host colonization, environmental persistence, and immune evasion [47]. The presence of such genes in SPBM3 suggests that plant-based foods contaminated with *Salmonella* may harbor strains with considerable zoonotic and clinical significance.

Furthermore, prophage analysis revealed five distinct regions (Figure 2). Two intact prophages—regions a and b—similar to *Salmonella* phages 118970_sal3 (NC_031940) and Fels-1 (NC_010391), were found on contig 1, spanning 39.9 Kb and 31.3 Kb, respectively. Additionally, questionable prophages—regions c and d—resembling *Escherichia* phage 500465-1 (51.9 Kb) (NC_049342) and *Enterobacteria* phage P4 (35.0 Kb) (NC_001609) were identified on contigs 3 and 5, respectively. Another intact prophage, located in region e, similar to *Burkholderia cenocepacia* phage BcepMu (18.1 Kb) (NC_005882), was also identified (Table 2). Although AMR-associated genes were not detected within these prophage regions, several prophages carried potential virulence genes linked to bacterial fitness: prophage region b contained *pagK*, *pagO*, and *sopE2*, which encode invasion-associated effectors; prophage region c included *clpB*, *fljA*, and *smpB*, which enhance cellular survival under stress conditions; prophage region d had *idnK*, and prophage region e harbored *gtrA*, virulence genes reported in *Salmonella* long-term systemic infection [48,49,50].

Antimicrobial resistance (AMR) gene analysis of the SPBM3 genome consists of 66 putative AMR genes (Table 3 and Table S4), including multiple multidrug efflux pump systems such as *acrAB*, *acrEF*, *mdtK*, and *marA*, as well as polymyxin resistance determinants like *pmrCEF*. Many of these genes are widely distributed among *Salmonella* populations and have significant implications for both human and animal health, as their presence may facilitate the development of resistance, particularly under antibiotic exposure. Efflux pump mechanisms, especially those belonging to the Resistance–Nodulation–Division (RND) superfamily, play a major role in mediating multidrug resistance by exporting a broad spectrum of antibiotics out of Gram-negative bacterial cells.

In *Salmonella*, systems such as AcrAB–TolC are regulated by global transcription factors including RamA, MarA, SoxS, and BaeR, which respond to environmental cues such as bile salts, indole, and antibiotics, thereby enabling rapid adaptation and a reduction in intracellular drug concentrations [51]. Overexpression of these efflux pumps can lead to elevated resistance levels and is also associated with increased biofilm formation and virulence [52]. In addition, the presence of *pmrCEF* genes highlights another adaptive resistance mechanism. These genes are regulated by the PmrA/PmrB two-component system, enabling post-translational modifications of lipid A (such as the addition of phosphoethanolamine), which reduce the binding efficacy of polymyxins like colistin [53]. However, such modifications are typically induced only under certain environmental pressures, such as low Mg^2+^, acidic pH, or the presence of antimicrobial peptides, and may not manifest phenotypically unless the regulatory cascade is activated [54].

It is important to note that the identification of virulence and AMR genes in the genome does not necessarily equate to functional pathogenicity. While these findings highlight potential risks, confirmation would require additional studies such as cell invasion assays, animal infection models, or dose–response assessments. Our study was designed to provide genomic evidence as a first step toward evaluating the safety of PBM products. Future investigations, potentially focusing on pathogenesis, should explore the functional aspects of these genes to fully assess their impact on human health.

### 3.3. Antibiotic Susceptibility Test

The antibiotic susceptibility test of *S.* Anatum SPBM3 revealed that the isolate was susceptible to a broad range of antibiotics, as shown in Table 4. However, the isolate showed intermediate to three antibiotics: streptomycin, ciprofloxacin, and colistin. There was no resistance profile observed.

Despite the extensive genetic reservoir for resistance in SPBM3, phenotypic testing showed that this strain remained susceptible to most antibiotics, with only intermediate resistance observed for streptomycin, ciprofloxacin, and colistin (Table 3). This genotype–phenotype discrepancy, which has been reported in other studies as well, may result from tight regulatory control of AMR gene expression, the absence of inducing environmental conditions during laboratory susceptibility testing, or limitations in current phenotypic assays that fail to detect subtle resistance phenotypes [52]. Importantly, the presence of these AMR genes, even in the absence of observable resistance, poses a latent threat—exposure to antibiotic pressure or specific environmental triggers could rapidly drive SPBM3 toward a resistant phenotype.

## 4. Conclusions

This study investigated the occurrence, genomic traits, and antibiotic susceptibility of *Salmonella* in plant-based meat (PBM) products. We detected *S.* Anatum ST64 in one raw sample (1.59%), whose genome carried diverse virulence genes, prophages, and antimicrobial resistance determinants, while showing intermediate resistance to streptomycin, ciprofloxacin, and colistin. These findings highlight potential microbial threats in PBM products and the need to dispel assumptions of inherent safety. Future food safety surveillance should integrate whole-genome sequencing and adopt a One Health perspective. We recommend strengthened hygiene practices, targeted monitoring, and inclusion of PBM in national microbial risk assessments to ensure consumer safety as the PBM market expands.

## Figures and Tables

**Table 1:** Prevalence of *Salmonella* in plant-based meat products collected from the local retail market.

Samples *	Types of Meat Analog	Positive Samples/Total Sample (% Prevalence)
Raw PBM	Ground pork analog	1/28 (3.57)
(*n* = 41)	Mushroom meat	0/2
	Plant-based burger	0/11
	Total	1/41 (2.44)
Cooked PBM	Chicken tender analog	0/14
(*n* = 22)	Chicken breast analog	0/2
	Nugget analog	0/2
	Beef-style strip	0/4
	Total	0/22 (0.0)
Total		1/63 (1.59)

* Plant-based meat products collected from local retail markets in Bangkok, Thailand.

**Table 2:** Prophage regions of *Salmonella* Anatum SPBM3 isolated from plant-based meat.

Region	Region Length (Kb)	Total Proteins	Region Position	Most Common Phage	%GC
a	39.9	56	100,364–140,281	PHAGE_Salmon_118970_sal3	50.13
b	31.3	27	328,604–359,952	PHAGE_Salmon_Fels_1	48.15
c	37.6	27	497,528–549,520	PHAGE_Escher_500465_1	53.38
d	35.0	14	159,972–195,020	PHAGE_Entero_P4	48.71
e	18.1	22	462,691–480,845	PHAGE_Burkho_BcepMu	51.29

**Table 3:** Putative antibiotic resistance genes found in *Salmonella* Anatum SPBM3.

Source ID	Gene	Product
YP_492255.1	*PmrB*	Sensor protein BasS/PmrB (activates BasR/PmrA)
NP_417177.1	*alaS*	Alanyl-tRNA synthetase
NP_415791.1	*cysB*	Cys regulon transcriptional activator CysB
NP_415222.1	*kdpE*	DNA-binding response regulator KdpE
AAF03531.1	*AAC(6′)-Iy*	Aminoglycoside N(6′)-acetyltransferase
CDO13981.1	*PhoP*	Transcriptional regulatory protein PhoP
AAC74603.2	*marR*	Multiple antibiotic resistance protein MarR
NP_458564.1	*soxR*	Redox-sensitive transcriptional activator SoxR
NP_414997.1	*acrR*	Transcriptional regulator of acrAB operon, AcrR
ACH50230.1	*ramR*	Transcriptional regulator, AcrR family
NP_463130.1	*soxS*	DNA-binding transcriptional dual regulator SoxS
AAA50993.1		Translation elongation factor Tu
AAA50993.1		Translation elongation factor Tu
AAA50993.1		Translation elongation factor Tu
AAA50993.1		Translation elongation factor Tu
NP_461214.1	*gyrA*	DNA gyrase subunit A
NP_462735.1	*gyrB*	DNA gyrase subunit B
NP_462089.1	*parC*	DNA topoisomerase IV subunit A
NP_462096.1	*parE*	DNA topoisomerase IV subunit B
CDJ72593	*GlpT*	Glycerol-3-phosphate transporter
CDJ73208	*UhpT*	Hexose phosphate transport protein UhpT
AIL15701	*murA*	UDP-N-acetylglucosamine 1-carboxyvinyltransferase
NP_415372.1	*nfsA*	Oxygen-insensitive NADPH nitroreductase
NP_312937.1	*rpoB*	DNA-directed RNA polymerase beta subunit
YP_491362.1	*folP*	Dihydropteroate synthase
NP_415632.1	*mfd*	Transcription-repair coupling factor
NP_416715.1	*YojI*	ABC-type siderophore export system, fused ATPase and permease components
YP_490697.1	*acrD*	Aminoglycosides efflux system AcrAD-TolC, inner-membrane proton/drug antiporter AcrD (RND type)
NP_415434.1	*msbA*	Lipid A export permease/ATP-binding protein MsbA
AFH35853.1	*mdfA*	Multidrug efflux pump MdfA/Cmr (of MFS type), broad spectrum
AAC77293.1	*mdtM*	Multidrug efflux pump MdtM (of MFS type)
ABG77966.1	*acrB*	Multidrug efflux system AcrAB-TolC, inner-membrane proton/drug antiporter AcrB (RND type)
ABG77965.1	*acrA*	Multidrug efflux system AcrAB-TolC, membrane fusion component AcrA
AAC76298.1	*acrF*	Multidrug efflux system AcrEF-TolC, inner-membrane proton/drug antiporter AcrF (RND type)
AAC76297.1	*acrE*	Multidrug efflux system AcrEF-TolC, membrane fusion component AcrE
AAC76297.1	*acrE*	Multidrug efflux system AcrEF-TolC, membrane fusion component AcrE
AAC75733.1	*emrB*	Multidrug efflux system EmrAB-OMF, inner-membrane proton/drug antiporter EmrB (MFS type)
BAA16547.1	*emrA*	Multidrug efflux system EmrAB-OMF, membrane fusion component EmrA
AAC75136.1	*mdtB*	Multidrug efflux system MdtABC-TolC, inner-membrane proton/drug antiporter MdtB (RND type)
AAC75137.1	*mdtC*	Multidrug efflux system MdtABC-TolC, inner-membrane proton/drug antiporter MdtC (RND type)
AAC75135.2	*mdtA*	Multidrug efflux system MdtABC-TolC, membrane fusion component MdtA
NP_459346.1	*mdsB*	Multidrug efflux system, inner membrane proton/drug antiporter (RND type)
NP_459347.2	*mdsA*	Multidrug efflux system, membrane fusion component
NP_459345.2	*mdsC*	Multidrug efflux system, outer membrane factor lipoprotein of OprM/OprM family
YP_489321.1	*mdtG*	Multidrug resistance protein MdtG
AAC74149.2	*mdtH*	Multidrug resistance protein MdtH
NP_417544.5	*patA*	Putrescine aminotransferase
AAC75138.1	*mdtD*	Uncharacterized transporter MdtD of major facilitator superfamily (MFS)
AML99881.1	*mdtK*	Uncharacterized transporter YeeO
NP_416340.1	*mgrB*	PhoP/PhoQ regulator MgrB
NP_312864.1	*cpxA*	Copper sensory histidine kinase CpxA
NP_312865.1	*cpxR*	Copper-sensing two-component system response regulator CpxR
BAE77933.1	*CRP*	Cyclic AMP receptor protein
NP_309766.1	*H-NS*	DNA-binding protein H-NS
NP_417169.1	*emrR*	Multidrug resistance regulator EmrR (MprA)
NP_460903.1	*sdiA*	N-(3-oxohexanoyl)-L-homoserine lactone-binding transcriptional activator @ N-(3-oxooctanoyl)-L-homoserine lactone-binding transcriptional activator
YP_490321.1	*baeR*	Response regulator BaeR
BAA15934.1	*baeS*	Sensory histidine kinase BaeS
NP_459349.1	*golS*	Transcriptional regulator, MerR family
YP_489794.1	*marA*	Multiple antibiotic resistance protein MarA
AFK13828.1	*ramA*	Transcriptional activator RamA
BAE78116.1	*PmrC*	Lipid A phosphoethanolamine transferase EptA/PmrC
AAC75315.1	*arnA*	UDP-4-amino-4-deoxy-L-arabinose formyltransferase/UDP-glucuronic acid oxidase (UDP-4-keto-hexauronic acid decarboxylating)
AAC75089.1	*PmrE*	UDP-glucose 6-dehydrogenase
AAC75314.1	*PmrF*	Undecaprenyl-phosphate 4-deoxy-4-formamido-L-arabinose transferase
AAC76093.1	*bacA*	Undecaprenyl-diphosphatase
NP_414618.4	*leuO*	LysR family transcriptional activator LeuO

**Table 4:** Antibiotic susceptibility test of *Salmonella* Anatum SPBM3.

Antibiotics	Diameter Zone (mm)	Interpretation
Ampicillin	25	S
Amoxicillin-Clavulanic acid	26	S
Gentamicin	16	S
Streptomycin	12	I
Ceftriaxone	29	S
Tetracycline	19	S
Ciprofloxacin	29	I
Nalidixic acid	22	S
Trimethoprim-Sulfamethoxazole	24	S
Chloramphenicol	26	S
Colistin	12	I
Tobramycin	16	S
Amikacin	20	S

S: susceptible, I: intermediate.

## Data Availability

All data supporting the findings of this study are available within the paper and its Supplementary Information. This Whole Genome Shotgun project has been deposited at DDBJ/ENA/GenBank under the accession JBQJIX000000000.

## Supplementary Materials

The following supporting information can be downloaded at: https://www.mdpi.com/article/10.3390/foods14213710/s1, Table S1: Composition of plant-based meat products analyzed in this study. Table S2: ANIb calculation; Table S3: Putative virulence genes; Table S4: Putative AMR genes; Supplemental Data S1: Metadata for each bacteria used in the phylogenetic analysis.

## References

[1] Smetana S., Mathys A., Knoch A., Heinz V. (2015). Meat alternatives: Life cycle assessment of most known meat substitutes. Int. J. Life Cycle Assess..

[2] Kumar P., Chatli M.K., Mehta N., Singh P., Malav O.P., Verma A.K. (2021). Meat analogues: Health promising sustainable meat substitutes. Crit. Rev. Food Sci. Nutr..

[3] Sha L., Xiong Y.L. (2020). Plant protein-based alternatives of reconstructed meat: Science, technology, and challenges. Trends Food Sci. Technol..

[4] Boye J.I., Arcand Y. (2013). Current trends in green technologies in food production and processing. Food Eng. Rev..

[5] Boukid F., Castellari M., Vegarud G.E., Sagué M.S. (2021). Recent advances in plant-based meat analogues: Raw materials, technologies, and challenges. Curr. Opin. Food Sci..

[6] *Salmonella* (Non-Typhoidal). https://www.who.int/news-room/fact-sheets/detail/salmonella-(non-typhoidal).

[7] Shoaib M., Xu J., Meng X., Wu Z., Hou X., He Z., Shang R., Zhang H., Pu W. (2023). Molecular epidemiology and characterization of antimicrobial-resistant *Staphylococcus haemolyticus* strains isolated from dairy cattle milk in Northwest, China. Front. Cell. Infect. Microbiol..

[8] Parker E.M., Parker A.J., Short G., O’Connor A.M., Wittum T.E. (2022). *Salmonella* detection in commercially prepared livestock feed and the raw ingredients and equipment used to manufacture the feed: A systematic review and meta-analysis. Prev. Vet. Med..

[9] Carrasco E., Morales-Rueda A., García-Gimeno R.M. (2012). Cross-contamination and recontamination by *Salmonella* in foods: A review. Food Res. Int..

[10] Bartula K., Begley M., Latour N., Callanan M. (2023). Growth of food-borne pathogens *Listeria* and *Salmonella* and spore-forming *Paenibacillus* and *Bacillus* in commercial plant-based milk alternatives. Food Microbiol..

[11] Allard M.W., Strain E., Melka D., Bunning K., Musser S.M., Brown E.W., Timme R. (2016). Practical value of food pathogen traceability through building a whole-genome sequencing network and database. J. Clin. Microbiol..

[12] Ronholm J., Nasheri N., Petronella N., Pagotto F. (2016). Navigating microbiological food safety in the era of whole-genome sequencing. Clin. Microbiol. Rev..

[13] How Does Whole Genome Sequencing Work?. https://www.cdc.gov/pulsenet/php/wgs/index.html.

[14] (2017). Microbiology of the Food Chain—Horizontal Method for the Detection, Enumeration and Serotyping of Salmonella—Part 1: Detection of *Salmonella* spp.

[15] Andrews S. FastQC: A Quality Control Tool for High Throughput Sequence Data. https://www.bioinformatics.babraham.ac.uk/projects/fastqc/.

[16] Chen S., Zhou Y., Chen Y., Gu J. (2018). fastp: An Ultra-Fast All-in-One FASTQ Preprocessor. Bioinformatics.

[17] Richter M., Rosselló-Móra R., Glockner F.O., Peplies J. (2015). JSpeciesWS: A web server for prokaryotic species circumscription based on pairwise genome comparison. Bioinfomatics.

[18] Jolley K.A., Bray J.E., Maiden M.C. (2018). Open-access bacterial population genomics: BIGSdb software, the PubMLST.org website and their applications. Wellcome Open Res..

[19] Seemann T. (2014). Prokka: Rapid prokaryotic genome annotation. Bioinformatics.

[20] Page A.J., Cummins C.A., Hunt M., Wong V.K., Reuter S., Holden M.T., Fookes M., Falush D., Keane J.A., Parkhill J. (2015). Roary: Rapid large-scale prokaryote pan genome analysis. Bioinformatics.

[21] Nguyen L.T., Schmidt H.A., Von Haeseler A., Minh B.Q. (2015). IQ-TREE: A fast and effective stochastic algorithm for estimating maximum-likelihood phylogenies. Mol. Biol. Evol..

[22] Letunic I., Bork P. (2024). Interactive Tree of Life (iTOL) v6: Recent updates to the phylogenetic tree display and annotation tool. Nucleic Acids Res..

[23] Arndt D., Grant J.R., Marcu A., Sajed T., Pon A., Liang Y., Wishart D.S. (2016). PHASTER: A better, faster version of the PHAST phage search tool. Nucleic Acids Res..

[24] Zhou Y., Liang Y., Lynch K.H., Dennis J.J., Wishart D.S. (2011). PHAST: A fast phage search tool. Nucleic Acids Res..

[25] Wishart D.S., Han S., Saha S., Oler E., Peters H., Grant J.R., Stothard P., Gautam V. (2023). PHASTEST: Faster than PHASTER, better than PHAST. Nucleic Acids Res..

[26] Olson R.D., Assaf R., Brettin T., Conrad N., Cucinell C., Davis J.J., Dempsey D.M., Dickerman A., Dietrich E.M., Kenyon R.W. (2023). Introducing the Bacterial and Viral Bioinformatics Resource Center (BV-BRC): A resource combining PATRIC, IRD and ViPR. Nucleic Acids Res..

[27] Carattoli A., Zankari E., García-Fernández A., Voldby Larsen M., Lund O., Villa L., Aarestrup F.M., Hasman H. (2014). In silico detection and typing of plasmids using PlasmidFinder and plasmid multilocus sequence typing. Antimicrob. Agents Chemother..

[28] Clinical and Laboratory Standards Institute (CLSI) (2024). Performance Standards for Antimicrobial Susceptibility Testing.

[29] Bonaldo F., Avot B.J.P., De Cesare A., Aarestrup F.M., Otani S. (2023). Foodborne pathogen dynamics in meat and meat analogues analysed using traditional microbiology and metagenomic sequencing. Antibiotics.

[30] Canadian Food Inspection Agency (2023). Bacterial Pathogens and Indicators in Ready-to-Eat Non-Soy Plant-Based Meat Alternatives—April 1, 2020 to March 31, 2023 (Final Report).

[31] Yang X., Wu Q., Zhang J., Huang J., Chen L., Wu S., Zeng H., Wang J., Chen M., Wu H. (2019). Prevalence, bacterial load, and antimicrobial resistance of *Salmonella* serovars isolated from retail meat and meat products in China. Front. Microbiol..

[32] Santos P.D.M., Widmer K.W., Rivera W.L. (2020). PCR-based detection and serovar identification of *Salmonella* in retail meat collected from wet markets in Metro Manila, Philippines. PLoS ONE.

[33] Sun T., Liu Y., Qin X., Aspridou Z., Zheng J., Wang X., Li Z., Dong Q. (2021). The prevalence and epidemiology of *Salmonella* in retail raw poultry meat in China: A systematic review and meta-analysis. Foods.

[34] Feasey N.A., Dougan G., Kingsley R.A., Heyderman R.S., Gordon M.A. (2012). Invasive non-typhoidal salmonella disease: An emerging and neglected tropical disease in Africa. Lancet.

[35] Rasko D.A., Rosovitz M.J., Myers G.S., Mongodin E.F., Fricke W.F., Gajer P., Crabtree J., Sebaihia M., Thomson N.R., Chaudhuri R. (2008). The pangenome structure of *Escherichia coli*: Comparative genomic analysis of *E. coli* commensal and pathogenic isolates. J. Bacteriol..

[36] Hai D., Guo B., Qiao M., Jiang H., Song L., Meng Z., Huang X. (2023). Evaluating the potential safety risk of plant-based meat analogues by analyzing microbial community composition. Foods.

[37] Tóth A.J., Dunay A., Battay M., Illés C.B., Bittsánszky A., Süth M. (2021). Microbial spoilage of plant-based meat analogues. Appl. Sci..

[38] Gomes E., Araújo D., Nogueira T., Oliveira R., Silva S., Oliveira L.V., Azevedo N.F., Almeida C., Castro J. (2025). Advances in whole genome sequencing for foodborne pathogens: Implications for clinical infectious disease surveillance and public health. Front. Cell. Infect. Microbiol..

[39] Pornsukarom S., Van Vliet A.H., Thakur S. (2018). Whole genome sequencing analysis of multiple *Salmonella* serovars provides insights into phylogenetic relatedness, antimicrobial resistance, and virulence markers across humans, food animals and agriculture environmental sources. BMC Genom..

[40] Pornsukarom S., Tongthainan D., Phromwat P., Wannaratana S., Nakbubpa K., Muangsri S. (2025). Molecular epidemiology and antimicrobial resistance of *Salmonella* at the human–macaque–environment interface in Thailand: A One Health surveillance study. Vet. World.

[41] World Health Organization (2018). Whole Genome Sequencing for Foodborne Disease Surveillance: Landscape Paper.

[42] Destoumieux-Garzón D., Mavingui P., Boetsch G., Boissier J., Darriet F., Duboz P., Fritsch C., Giraudoux P., Roux F.L., Morand S. (2018). The one health concept: 10 years old and a long road ahead. Front. Vet. Sci..

[43] Karki H.S., Ham J.H. (2014). The roles of the shikimate pathway genes, *aroA* and *aroB*, in virulence, growth and UV tolerance of *Burkholderia glumae* strain 411gr-6. Mol. Plant Pathol..

[44] Roberts C.A., Al-Tameemi H.M., Mashruwala A.A., Rosario-Cruz Z., Chauhan U., Sause W.E., Torres V.J., Belden W.J., Boyd J.M. (2017). The Suf iron-sulfur cluster biosynthetic system is essential in *Staphylococcus aureus*, and decreased Suf function results in global metabolic defects and reduced survival in human neutrophils. Infect. Immun..

[45] Ishiguro N., Izawa H., Shinagawa M., Shimamoto T., Tsuchiya T. (1992). Cloning and nucleotide sequence of the gene (*citC*) encoding a citrate carrier from several *Salmonella* serovars. J. Biol. Chem..

[46] Burns D.M., Burger M.J., Beacham I.R. (1995). Silent genes in bacteria: The previously designated ‘cryptic’ *ilvHI* locus of ‘*Salmonella typhimurium* LT2’ is active in natural isolates. FEMS Microbiol. Lett..

[47] Kabir A., Kelley W.G., Glover C., Erol E., Helmy Y.A. (2025). Phenotypic and genotypic characterization of antimicrobial resistance and virulence profiles of *Salmonella enterica* serotypes isolated from necropsied horses in Kentucky. Microbiol. Spectr..

[48] Wahl A., Battesti A., Ansaldi M. (2019). Prophages in Salmonella enterica: A driving force in reshaping the genome and physiology of their bacterial host?. Mol. Microbiol..

[49] Andrews K., Landeryou T., Sicheritz-Pontén T., Nale J.Y. (2024). Diverse prophage elements of *Salmonella enterica* serovars show potential roles in bacterial pathogenicity. Cells.

[50] Trofeit L., Sattler E., Künz J., Hilbert F. (2023). *Salmonella* prophages, their propagation, host specificity and antimicrobial resistance gene transduction. Antibiotics.

[51] Nikaido E., Yamaguchi A., Nishino K. (2008). AcrAB multidrug efflux pump regulation in *Salmonella enterica* serovar Typhimurium by RamA in response to environmental signals. J. Biol. Chem..

[52] Alenazy R. (2022). Antibiotic resistance in *Salmonella*: Targeting multidrug resistance by understanding efflux pumps, regulators and the inhibitors. J. King Saud. Univ. Sci..

[53] Lee H., Hsu F.F., Turk J., Groisman E.A. (2004). The PmrA-regulated pmrC gene mediates phosphoethanolamine modification of lipid A and polymyxin resistance in *Salmonella enterica*. J. Bacteriol..

[54] Kawasaki K., China K., Nishijima M. (2007). Release of the lipopolysaccharide deacylase PagL from latency compensates for a lack of lipopolysaccharide aminoarabinose modification-dependent resistance to the antimicrobial peptide polymyxin B in *Salmonella enterica*. J. Bacteriol..

