# Supplementary material for: Detection, Genomic Characterization, and Antibiotic Susceptibility of Salmonella Anatum SPBM3 Isolated from Plant-Based Meat
Source: Foods. 2025 Oct 30;14(21):3710. doi: 10.3390/foods14213710 (PMC12607527; doi:10.3390/foods14213710)
Supplement: Supplementary file 1 [file foods-14-03710-s001.zip › Table S1.pdf]

Table S1 Composition of plant-based meat products analyzed in this study.

Product type	Sample count (n)	Main protein source	Other major ingredients
Ground pork analog	28	Soy, Pea	Wheat gluten, canola oil, seasonings
Chicken tender analog	14	Soy	Coconut oil, wheat gluten, flavoring
Chicken breast analog	2	Mycoprotein, Pea	Sunflower oil, starch
Nugget analog	2	Soy	Wheat flour, spices
Beef-style strips	4	Pea	Canola oil, soy sauce, caramel color
Mushroom-based burger	2	Mushroom, Soy	Rice flour, coconut oil
Plant-based burger	11	Pea	Canola oil, coloring
